# Viral RNA Silencing Suppression: The Enigma of Bunyavirus NSs Proteins

**DOI:** 10.3390/v8070208

**Published:** 2016-07-23

**Authors:** Marcio Hedil, Richard Kormelink

**Affiliations:** Laboratory of Virology, Department of Plant Sciences, Wageningen University, Wageningen, 6708PB, The Netherlands; marcio.hedil@outlook.com

**Keywords:** RNAi, RNA silencing, innate immunity, bunyavirus, NSs, tospovirus, orthobunyavirus, RNA silencing suppression

## Abstract

The *Bunyaviridae* is a family of arboviruses including both plant- and vertebrate-infecting representatives. The *Tospovirus* genus accommodates plant-infecting bunyaviruses, which not only replicate in their plant host, but also in their insect thrips vector during persistent propagative transmission. For this reason, they are generally assumed to encounter antiviral RNA silencing in plants and insects. Here we present an overview on how tospovirus nonstructural NSs protein counteracts antiviral RNA silencing in plants and what is known so far in insects. Like tospoviruses, members of the related vertebrate-infecting bunyaviruses classified in the genera *Orthobunyavirus*, *Hantavirus* and *Phlebovirus* also code for a NSs protein. However, for none of them RNA silencing suppressor activity has been unambiguously demonstrated in neither vertebrate host nor arthropod vector. The second part of this review will briefly describe the role of these NSs proteins in modulation of innate immune responses in mammals and elaborate on a hypothetical scenario to explain if and how NSs proteins from vertebrate-infecting bunyaviruses affect RNA silencing. If so, why this discovery has been hampered so far.

## 1. Introduction—The Family of *Bunyaviridae*

**Figure 1:**
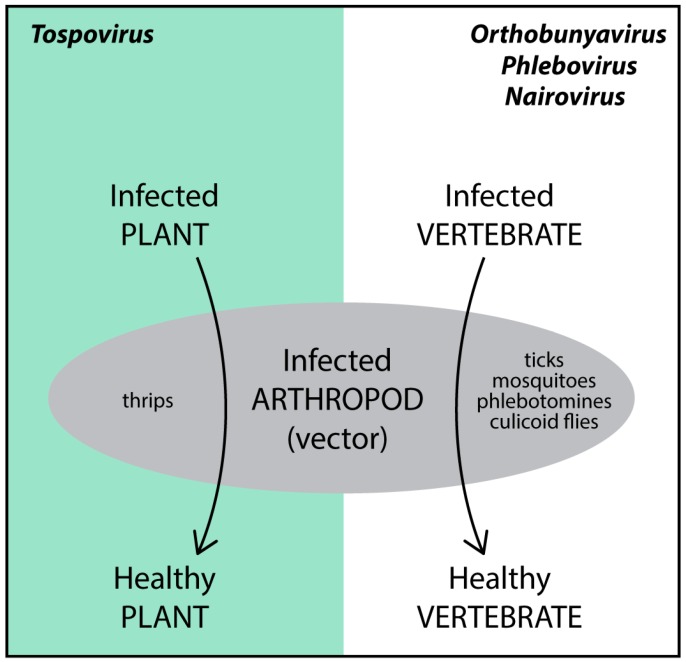
Bunyaviruses and their arthropod vectors.

The *Bunyaviridae*, with more than 350 identified species, is divided in five genera and contains several important viruses that cause major problems in human/animal health and agriculture production systems. All five genera of this family contain viruses pathogenic to either animals/humans (*Orthobunyavirus*, *Phlebovirus*, *Nairovirus* and *Hantavirus*) or plants (*Tospovirus*). Most bunyaviruses are arthropod-borne viruses (arboviruses), as they replicate in the arthropods by which they are transmitted (Figure 1). Hantaviruses present an exception, as they are rodent-borne and no arthropod vector has been identified so far. 

**Figure 2:**
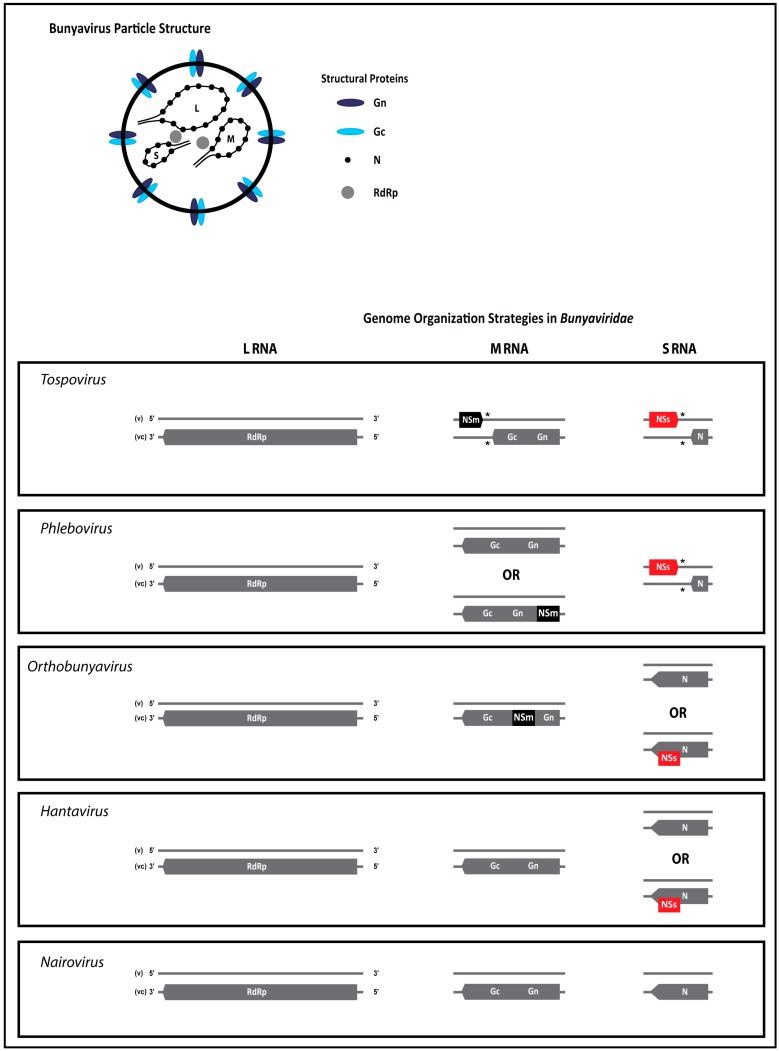
Particle structure and genome organization of bunyaviruses. L, M, and S RNA (large, medium, and small RNA segments, respectively), RdRP (viral RNA-dependent RNA polymerase), Gn and Gc (glycoproteins derived from the N-terminus and C-terminus of the precursor protein, respectively), NSm (non-structural protein of the M RNA), N (nucleocapsid protein), NSs (non-structural protein of the S RNA). (*) intergenic region (IGR).

Members of all five genera in the *Bunyaviridae* share several features. Bunyavirus particles are enveloped and generally spherical. Viral glycoproteins are embedded in the envelope membrane and presented as spikes on the outside. The core of virus particles contains the single-stranded (ss)RNA genome that is encapsidated by a nucleocapsid (N) protein and small amounts of the viral RNA-dependent RNA polymerase (RdRp, also denoted L protein). The bunyavirus RNA genome is tripartite and segments have either a negative or ambisense polarity (Figure 2). Genome organization strategies vary among members of different genera and may diversify even among members within a genus, as observed with orthobunyaviruses and phleboviruses. In general, though, the bunyavirus genome codes for four structural and up to two non-structural proteins. The L RNA is of complete negative polarity and contains a single open reading frame (ORF) on the viral complementary (vc)RNA that encodes the RdRp. With the exception of tospoviruses, the M RNA of all other bunyaviruses is of negative polarity and contains one single ORF on the vc-strand coding for the precursor to the two glycoproteins (Gn and Gc), and in a few cases an additional non-structural protein NSm. The M RNA of tospoviruses contains an ambisense gene arrangement, and encodes a NSm on the viral (v)RNA strand and the glycoprotein precursor on the vcRNA. Tospovirus NSm protein facilitates the movement of viral ribonucleoproteins (RNPs) from cell-to-cell and presents an adaptation of this group of viruses to plants as a host. The S RNA segment is of negative polarity for members of the genera *Orthobunyavirus*, *Hantavirus* and *Nairovirus*, or ambisense for members of the genera *Phlebovirus* and *Tospovirus* [1,2,3,4,5,6]. The negative polarity S RNA encodes the major structural N protein on the vcRNA strand and, in certain members of orthobunyaviruses and hantaviruses, an additional non-structural protein (NSs) in an overlapping reading frame. For members of genera with ambisense S RNA, the NSs protein is encoded, separate from the *N* gene, by a second non-overlapping ORF on the vRNA strand (Figure 2).

During their infection cycle, all viruses encounter the host innate immune system as one of the first lines of host defense. In response to that, viruses have evolved various strategies to counteract the host innate immune system. In the case of bunyaviruses, the NSs protein is a knowingly important modulator of host innate immune responses, and a virulence factor [1,2,3,4,5,6,7]. In vertebrates, the interferon (IFN) pathway plays a major role in antiviral defense, and accordingly IFN-antagonist activity is found in the NSs of vertebrate-infecting bunyaviruses (reviewed by [7]). In plants and arthropods, where IFN pathway is not present, RNA silencing is an important part of antiviral innate immunity, and during infection of plants (by tospoviruses) and arthropods (by arthropod-borne bunyaviruses), members of the *Bunyaviridae* are targeted by antiviral RNA silencing. However, so far only the NSs protein of tospoviruses has been irrefutably demonstrated to have RNA silencing suppression (RSS also known as viral suppressors of silencing, VSRs) activity [8,9,10], which is relevant for the establishment of a successful infection of plant hosts. Concerning the arthropod vector, information related to the possible effect of NSs on antiviral RNA silencing during infection in arthropods remains scarce for both plant- and vertebrate-infecting bunyaviruses likewise. In this review we will present the state of the art on the modulation of host defense responses by bunyavirus NSs proteins, with emphasis on its effect on antiviral RNA silencing, to finally discuss the enigma surrounding NSs from bunyaviruses and its (possible) effect on arthropod and mammalian antiviral RNA silencing.

## 2. Antiviral RNA Silencing 

**Figure 3:**
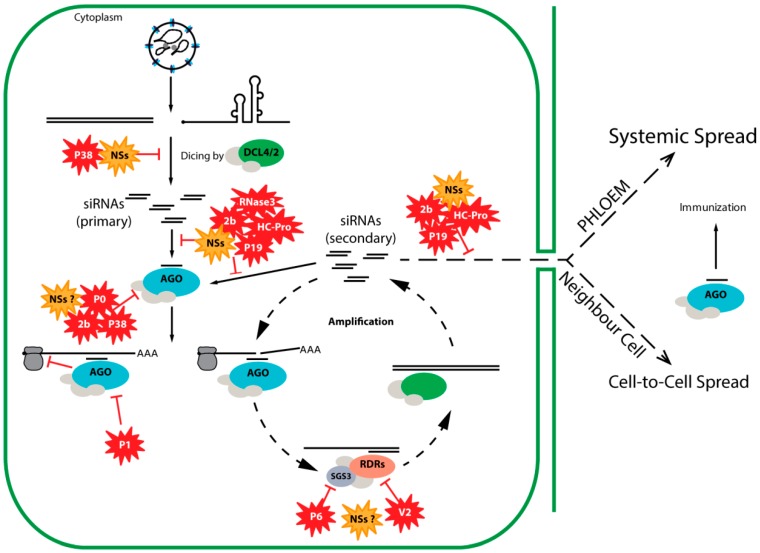
Schematic representation of plant antiviral RNA silencing and the counter-strategies used by several viral RSS proteins (indicated by the red symbols and, in the case of tospovirus NSs, orange symbols). AGO: argonaute protein; DCL: dicer-like protein; RDR: host-encoded RNA-dependent RNA polymerase; SGS3: protein suppressor of gene silencing 3; HC-Pro: helper component-proteinase; siRNAs: small interfering RNAs.

RNA silencing (also known as RNA interference, RNAi) is a gene regulatory mechanism conserved among eukaryotic organisms. In plants, fungi and invertebrates an antiviral RNA silencing pathway is triggered by double-stranded (ds)RNA structures (viral dsRNA intermediates, intramolecular hairpin structures in viral ssRNA, complementary viral transcripts) that arise during viral infections [11]. These viral dsRNA molecules are recognized and processed by Dicer enzymes (members from the RNase III family) into viral small interfering RNAs (vsiRNAs) approximately 21 nt in size [12,13]. From these, one strand is loaded in an argonaute (AGO) protein, the effector component of the RNA-induced silencing complex (RISC) [14,15]. The siRNA-programmed RISC surveils the cell for the presence of (viral) RNA target sequences with siRNA-sequence complementarity and next through AGO slicer activity cleaves the RNA target, initiating its degradation (Figure 3). Plants, fungi, worms, but not insects or mammals, contain RNA-dependent RNA polymerases (RDRs), which are essential components for amplification of the silencing signal [16,17]. As a result of amplification, secondary siRNAs are produced, leading to a more robust silencing response (Figure 3). Secondary siRNAs are important components of the systemic silencing response and travel short and long distances, in plants, respectively, through plasmodesmata (to neighbouring cells) and phloem (to distant parts of the plant). In the plant model *Arabidopsis thaliana*, the proteins involved in the amplification process include, among others, dicer-like proteins (DCLs) (processing also the de novo dsRNA in secondary siRNAs), cellular RDRs (synthesis of de novo dsRNA), and protein suppressor of gene silencing 3 (SGS3, a cofactor of RDR6) [18].

Although RNA silencing is a conserved eukaryotic mechanism, diversification occurred during evolution of the species, with the number of components varying among different organisms. *A. thaliana*, one of the best described (plant) models for RNA silencing, has four DCLs (DCL1–4), ten AGOs (AGO1–10) and six RDRs (RDR1–6). Some of these proteins have partially redundant roles, but specific combinations are known to be involved in the different plant RNA silencing pathways. For a more detailed review on this matter, readers are referred to [19]. While RNA silencing has been demonstrated to act antiviral in plants, fungi and invertebrates more than a decade ago, it was not until 2013 that studies involving analysis of RNA silencing in mammalian undifferentiated cells as well as the analysis of “in phase” production of siRNAs elegantly showed the presence of antiviral RNA silencing also in vertebrates [20,21].

## 3. Viral Strategies to Evade Plant Antiviral RNA Silencing Defense

In order to successfully infect hosts that contain an active antiviral RNA silencing defense system, viruses have evolved diverse strategies. Many of these have been identified during extensive studies on a wide range of plant viruses. Brome mosaic virus (BMV) replicates inside endoplasmic reticulum (ER)-derived spherules [22], therefore avoiding being perceived by the RNA silencing machinery. Other viruses, such as cauliflower mosaic virus (CaMV), during their infection produce decoy-RNA (generated from noncoding regions) that is targeted by Dicer, hence diverting the host RNA silencing machinery from targeting essential regions of the viral genome and transcripts [23].

A strategy that appears to be one of the most common among (plant) viruses is the expression of RSS proteins with the ability to suppress RNA silencing by targeting important steps of this antiviral pathway. These viral proteins are structurally and functionally diverse and employ a myriad of strategies to target key components of the RNA silencing pathway (Table 1) [18]. Due to the large number of RSS proteins already identified, it would be too extensive to describe each of them individually. Therefore, here only the most well known strategies used by viral RSS will be explained. More in depth reviews are available in literature [19,24].

A strategy used by many RSS is the binding and sequestration of dsRNA. Binding to dsRNA can be limited by the size of the molecule and some RSS proteins are able to bind only siRNAs. Size-selective binding to siRNAs has been shown for, e.g., P19 (carnation Italian ringspot virus, CIRV), P21 (beet yellows virus, BYV), γB (barley stripe mosaic virus, BSMV) and HC-Pro (tobacco etch virus, TEV) [47,48] (Figure 3, Table 1). Other RSS, such as aureusvirus P14 (pothos latent virus, PoLV) and carmovirus P38 (turnip crinkle virus, TCV), are not limited by the size of the dsRNA molecule, binding dsRNA size-independently and hence being able to not only sequester siRNAs, but also to prevent or interfere with DCL cleavage of long dsRNA triggers (Figure 3).

Another strategy of interference involves the inhibition of HEN1-dependent methylation of siRNAs (in plants and flies this methylation is necessary for stabilization of siRNA). Interference with methylation has been shown for HC-Pro (TEV) and P19 (CIRV) [63]. A more unusual siRNA-targeting strategy is performed by RNaseIII enzymes of some viruses, e.g., RNase3 of sweet potato chlorotic stunt virus (SPCSV, genus *Crinivirus*), which contains endonuclease activity and cleaves siRNAs into inactive smaller products which are unable to activate RISC [45,46]. Several RSS achieve suppression of RNA silencing by preventing RISC assembly through targeting of argonaute proteins. Through GW-WG motifs (AGO-hooks) RSS proteins, such as P38 (TCV), are able to interact with and compromise argonaute activity [71]. Some RSS—P38 (TCV), P19 (CymRSV), HC-Pro (TEV), 2b (CMV)—modulate accumulation of miR168 (regulates AGO1 mRNA), consequently interfering with AGO1-related silencing [76]. Another strategy is targeting AGO1 for degradation, described for poleroviral P0 [55]. While most RSS do not effectively target AGO1 once it is assembled into RISC, P1 (SPMMV) is able to inhibit programmed RISC, which it achieves by targeting AGO1 via GW-WG motifs present in its N-terminal region [62].

The amplification phase of RNA silencing is also targeted by several RSS. Interaction of V2 (TYLCV) with SGS3 (a cofactor of RDR6) was demonstrated to be essential for its RSS activity [25]. Nucleorhabdovirus P6 (RYSV) is an RSS that inhibits systemic (but not local) silencing and this is likely achieved by interaction with RDR6 and blocking of RDR6-mediated secondary siRNA synthesis [36].

## 4. Arthropod RNA Silencing and Arboviruses

After the discovery of RNA silencing in *Caenorhabditis elegans* and plants, one of the first evidence of natural antiviral silencing in insect species came in 2002 during studies on flock house virus (FHV) infection of *Drosophila melanogaster* cells, in which FHV B2 protein was identified as a RSS [82]. The presence of this (gene regulation) mechanism has also been shown in other arthropods, including flies (e.g., fruit fly), mosquitoes, spiders and ticks [83,84]. Drosophila is one of the insect species where RNA silencing is probably best characterized, and although the mechanism is similar to the one of plants, it also shows important differences. Drosophila contains two Dicer proteins, three Argonaute proteins and to date no RDR has been identified in insects (reviewed in [85]). In the case of antiviral RNA silencing Dicer-2 recognizes and cleaves dsRNA structures (generated during virus replication) into siRNAs, from which one strand activates a RISC complex containing AGO2 to surveil the cell for ssRNA with sequence complementarity to the siRNA [14,86,87,88]. The pathway also includes other proteins such as Loquacious (Loqs, which increases affinity of Dicer-2 to dsRNA), R2D2 (aids siRNA loading in AGO2 and RISC assembly), component 3 promoter of RISC (C3PO, facilitates AGO2 endonucleolytic cleavage of the siRNA passenger strand) and HEN1 (stabilizes the guide strand by methylating its 3′ terminal nucleotide) (a deeper review of arthropod RNA silencing can be found in [89,90,91]). Similar to plant-infecting viruses, several arthropod-infecting viruses have been shown to counteract arthropod antiviral RNA silencing by expressing RSS proteins that can target different steps of the RNA silencing machinery. B2 from drosophila-infecting nodavirus FHV, one of the first RSS identified in arthropods, binds dsRNA size-independently and inhibits Dicer cleavage [92], but also directly interacts with the PAZ domain of *Drosophila* Dicer proteins [93]. B2 from Wuhan nodavirus (WhNV) has also been shown to bind dsRNA (small and long) (reviewed in [94]). Another example is protein 1A from cricket paralysis virus (CrPV), which suppresses RNA silencing by directly interacting with AGO2 [95], while 1A from *Drosophila* C virus (DCV) binds dsRNA and blocks Dicer-2 processing of dsRNA into siRNAs but does not bind siRNAs [96].

Arthropods are vectors for viruses (arboviruses) from several families (including *Flaviviridae*, *Togaviridae*, *Reoviridae* and *Bunyaviridae*). During persistent-propagative transmission, both plant- and animal-infecting arboviruses replicate in the arthropod vector [97,98], following a similar route of infection. The primary infection site is the midgut epithelium and in order to further disseminate in the arthropod organism and reach the salivary glands the virus must pass the midgut barrier, a determinant factor for vector competence (reviewed in [97,98,99]). Several studies indicate that RNA silencing is a relevant part of the midgut barrier and plays an important role in arbovirus infection and vector competence [100,101]. However, the antiviral role of midgut RNA silencing remains under debate and may also vary according to the arthropod species, as a recent investigation of alphavirus infection in *Anopheles gambiae* mosquitoes presents evidence that RNA silencing is active however it does not seem to have antiviral impact on the initial midgut infection, but only at later stages of infection [102]. Supporting the importance of antiviral RNA silencing in modulation of arbovirus infection, in arthropods where RNA silencing has been compromised or suppressed (e.g., by the action of a strong RNA silencing suppressor), arboviruses replicate to higher titers and the infection becomes pathogenic [103,104], which indicates that arthropod RNA silencing also modulates the equilibrium of an arbovirus persistent infection of the vector. Some strategies used by arboviruses to modulate antiviral RNA silencing include decoy strategies, e.g., the flavivirus small structured non-coding RNA from the viral 3′UTR referred to as subgenomic flavivirus RNA (sfRNA) [105] and the nucleic acid mediated decoy mechanisms of alphaviruses [106].

Members of the *Bunyaviridae* are arboviruses, and as such replicate in both the host (plant or vertebrate) as well as in the arthropod vector. While infecting arthropods, bunyaviruses are targeted by RNA silencing, as illustrated by production of virus-specific siRNAs (Table 2) [107,108,109]. It has not yet been demonstrated whether arthropod infection by bunyavirus requires viral expression of a suppressor of arthropod RNA silencing. For plant-infecting tospoviruses, the NSs protein suppresses RNA silencing in plants [8,9] and this protein is expected to also modulate antiviral RNA silencing in thrips (although clear evidence for this is still needed). On the other hand, information on the effect of vertebrate-infecting bunyaviruses in either vertebrate or arthropod RNA silencing is scarce and limited to a few reports. Below, we review the current knowledge on the activity of NSs from bunyaviruses in the different environments where replication takes place. 

## 5. Tospovirus NSs Counter-Defense against the siRNA Branch of the Antiviral RNA Silencing Pathway in Plants

A first glimpse on the importance of NSs for tospoviral infection in plants came in the early 1990s, when elevated levels of NSs expression were observed to correlate to a higher virulence of tomato spotted wilt virus (TSWV) isolates and more severe symptoms [116]. About a decade later, the ability of NSs to counteract the RNA silencing defense mechanism in plants was demonstrated [8,9]. Further studies on tospoviral NSs (particularly from TSWV) revealed that it is able to bind small and long dsRNA [32,117], suggesting that NSs interferes in two important steps: by binding long dsRNA it prevents these molecules from being recognized and processed into siRNAs by Dicer-like proteins (DCLs), while by sequestering siRNAs it prevents RISC from being activated and programmed (Figure 3).

More recently it was also shown that NSs of TSWV, as well as those of groundnut ringspot virus (GRSV) and tomato yellow ring virus (TYRV), suppresses long-distance systemic silencing [10], which is likely a consequence from interference in the biogenesis of the systemic signal (siRNAs). Interestingly, this same study also shows that suppression of local and systemic silencing can be uncoupled, as TSWV NSs mutants which lost the ability to suppress local silencing were still able to suppress systemic silencing [10]. These observations suggest that, besides sequestration of long and short dsRNA, NSs possibly also interferes in a systemic silencing exclusive step downstream the siRNA biogenesis (Figure 3). Suppression of systemic silencing without affecting local silencing has been shown for other viral RSS proteins, such as nucleorhabdovirus P6, where it was suggested to block secondary siRNA production by interacting with RDR6 [36]. 

## 6. Tospovirus NSs Counter-Defense against the miRNA Branch of the Antiviral RNA Silencing Pathway in Plants

Next to the siRNA-mediated silencing, there is a branch of the RNA silencing pathway involving micro (mi)RNAs, which resemble siRNAs structurally and in size, but differ from those in their biogenesis, being processed from host-encoded transcripts that fold into imperfect stem-loop structures. The miRNA pathway plays a major role in regulation of eukaryotic endogenous gene expression, including expression of RNA silencing components. The best known example is the regulation of AGO1 expression by miR168 [118]. Interference with the miRNA pathway is also a viral strategy and several RSS proteins, such as tombusvirus P19 and cucumovirus 2b, are able to modulate miR168, causing a downregulation on AGO1 expression and this way affecting the antiviral RNA silencing response (Table 1).

Interference in the miRNA pathway has been described for TSWV NSs, shown to bind miRNAs in vitro, and to interfere with the miRNA pathway in planta [32]. In light of its affinity to long and small RNA duplex molecules, it is likely that TSWV interference in the miRNA pathway is accomplished at least partly through sequestration of miRNAs by NSs. Also for the GBNV NSs protein initial data indicate it interferes with the miRNA pathway [33]. 

## 7. TSWV NSs as Effector of Plant NLR-Mediated Intracellular Innate Immunity Response

Besides RNA silencing, another layer of the innate immunity system is represented by resistance genes and their R protein products. In plants the major class of dominant resistance (*R*) genes codes for the NB-LRR type, which are proteins consisting of three main domains. The N-terminal end is presented by a coiled-coil (CC) or Toll and interleukin-1 receptor (TIR) domain, followed by an internal nucleotide binding site (NBS) domain and a leucine-rich repeat (LRR) at the C-terminal end [119,120]. R proteins act as intracellular sensors of innate immunity and are highly pathogen specific. They are able to directly or indirectly perceive the presence of a pathogen by recognizing one of its effector proteins that often play a role in virulence and are referred to as avirulence factors (Avr). R proteins act like molecular switches that upon effector/Avr recognition trigger a resistance mechanism concomitant with a programmed cell death, leading to the appearance of small necrotic lesions (hypersensitive response, HR) at the site of pathogen entry. The establishment of HR prevents further dissemination of the pathogen throughout the plant host [121,122,123,124]. Some R proteins recognize viral RSS proteins, thereby acting as a plant counter-counter-defense against the viral counter-defense (RNA silencing suppression) against plant antiviral RNA silencing [125]. 

Recently, TSWV NSs protein was identified as the Avr for the single dominant *Tsw* resistance gene product, a protein that is also thought to belong to the class of NB-LRR genes [126]. In the constant battle between viruses and plants, viruses continuously keep on evolving and mutations on key amino acid residues of NSs lead to the appearance of so-called TSWV resistance-breaking (RB) isolates that do not trigger the *Tsw*-mediated HR response. Considering the role of NSs in counter defense against RNA silencing, mutations within this protein are likely fine-tuned to preserve (some) viral fitness and virulence, preventing virus clearance from the plant by antiviral RNA silencing. A study on engineered NSs mutants indicated that NSs RSS and Avr functions can be uncoupled. Furthermore, some of the resistance-breaking NSs mutants (both natural and engineered), mutated in predicted RNA-binding or putative Argonaute-binding domains, still suppress systemic silencing spread while having lost the ability to suppress local silencing [10,127]. Altogether, this supports the idea that the virus and its NSs protein can evolve to fine tune Avr and (local/systemic) RSS functions in order to lose (some of) its avirulence features while preserving a certain level of fitness [10,127].

## 8. Tospovirus NSs Counter Defense Against Antiviral RNA Silencing in Insects

Antiviral RNA silencing has already been demonstrated in several arthropods [96,128]. Also during an arbovirus infection of arthropod vectors the mechanism acts antiviral, more specifically in the midgut epithelium where RNA silencing seems to be an important part of the midgut barrier and influences viral dissemination to secondary tissues and vector competence [100]. In thrips, RNA silencing is functional and can be used as an investigative tool [129,130], however its role in antiviral defense has not been verified yet, and it remains to be investigated whether tospoviruses during propagative transmission by thrips are strongly targeted by antiviral RNA silencing and require a counter-defense by NSs. 

In contrast to the situation in plants, less is known about the function of tospovirus NSs in the insect vector (thrips). Evidence shows that TSWV NSs is expressed in cells of thrips *Frankliniella occidentalis* (western flower thrips, WFT), especially in midgut epithelial cells (primary infection site), associated muscle tissue and cells in the salivary glands, from where tospoviruses are transmitted to the plant during thrips feeding [131,132,133]. Although to date little information is available on the molecular modus operandi of NSs in the context of thrips infection, recent reports shed light on this matter and slowly a clearer picture is emerging on the importance of NSs during an arthropod infection.

The role of NSs as a transmission determinant in thrips was recently demonstrated in experiments with NSs-defective TSWV isolates which lost RSS activity and became non-transmissible by thrips. A closer look indicated that these viruses were still acquired by thrips larvae upon feeding on infected plants, however virus accumulation in adult thrips was severely reduced, which likely explains why adult thrips do not support transmission of these NSs-defective isolates upon a next probing-feeding attempt [134]. In light of the antiviral RNA silencing role in the midgut epithelium and its influence in vector competence observed for other arbovirus-arthropod vector interactions [100], it is likely that the observed reduction in vector competence and virus titers are linked to the lack of RSS activity in these NSs-defective isolates.

Although not in thrips, silencing suppression function of tospovirus NSs has been investigated in arthropods including mosquitoes (Diptera), caterpillar (Lepidoptera) and ticks. Two studies showed that TSWV NSs is able to suppress luciferase silencing, when expressed in mosquito cell lines (U4.4— *Aedes albopictus*) [110] and in tick cells (ISE6—*Ixodes scapularis*) [135]. In another study, TSWV NSs expressed from a recombinant baculovirus enhanced the replication of this recombinant virus to higher titers in *Spodoptera frugiperda* insect (lepidopteran) cell lines [136] and increased its virulence in caterpillar insects in vivo [137]. These observations support that NSs is also able to suppress antiviral RNA silencing in arthropods. 

There is indication that TSWV infection activates other thrips antiviral genes and defense responses—such as genes coding for antimicrobial peptides—proteins involved in pathogen recognition, receptors that activate innate immune response—such as Toll3—and members of signal transduction pathways activated by toll-like receptors [138]. Whether NSs has an effect in any of these other antiviral pathways in thrips is unknown. 

## 9. NSs from Vertebrate-Infecting Bunyaviruses—Antagonizing Mammalian Innate Immunity

Vertebrate-infecting viruses must deal with the innate immunity in their mammalian hosts, including the well-known IFN-based defense [139,140]. NSs from several vertebrate-infecting bunyaviruses are well reported for their IFN-antagonist activity. For orthobunyavirus Bunyamwera virus (BUNV), La Crosse virus (LACV) and phlebovirus Rift Valley fever virus (RVFV), the NSs protein inhibits the type I IFN system by blocking RNA polymerase II transcription, consequently leading to a shutoff of the antiviral response genes [110,141,142,143,144]. RVFV NSs has additionally been shown to induce specific degradation of dsRNA-dependent protein kinase (PKR) [145], a process that occurs independently from the NSs-mediated blocking of host gene transcription [146]. IFN-antagonistic activity has also been identified in the NSs of tick-borne phleboviruses, e.g., the NSs of Uukuniemi virus (UUKV) was shown to have weak IFN-antagonistic activity [147]. In the case of the tick-borne severe fever with thrombocytopenia syndrome virus (SFTSV), its NSs has been shown to form cytoplasmic inclusions involved in sequestration of host factors involved in RIG-I signaling as well as IFN signaling [148,149,150,151]. A more complete description of the diverse strategies employed by different phlebovirus NSs proteins to evade host IFN defense can be found in [152]. Several hantaviruses have been reported to contain, like orthobunyaviruses, an ORF overlapping the *N* gene and encoding a NSs protein with weak IFN-antagonistic properties [1]. For an extensive description on this subject readers are referred to excellent literature reviews [7,153]. In nairoviruses, a NSs protein has been identified only in Crimean-Congo hemorrhagic fever virus (CCHFV) and shown to induce apoptosis [154], however its effect on IFN pathway remains to be investigated.

Besides the IFN-induced innate immune responses, mammals contain additional layers of innate immunity that act against viruses, including antiviral RNA silencing [20,21]. Proteins from mammalian-infecting viruses have earlier been observed to possess RSS activity too, amongst which human immunodeficiency virus type 1 (HIV-1) trans-activator of transcription (Tat, Dicer interaction and inhibition), hepatitis C virus (HCV) core (Dicer interaction) and envelope E2 (AGO2 interaction), human influenza A NS1, Ebola virus VP35 and vaccinia virus E3L (all binding dsRNA), adenovirus VA (a non-coding RNA that folds into a stem loop structure and acts as a decoy for Dicer). Interestingly, all these very same viral proteins are known to act as IFN antagonists as well [155].

Thus far, only two papers have appeared on the identification of RSS activity in NSs proteins from vertebrate-infecting bunyaviruses in mammals, both with LACV NSs, but with contradictory outcomes. In one paper, RNA silencing was triggered by transfecting cells (human cell line 293T) with siRNAs, showing that in the additional presence of (transiently) over-expressed LACV NSs an apparent decrease of siRNA-triggered silencing was observed, which tempted the authors to suggest that LACV NSs exhibits RSS activity in mammals [156]. In another paper, researchers used LACV and recombinant LACVdelNSs viruses, and observed the outcome during infection in IFN-competent and IFN-deficient mammalian cell cultures (mouse embryo fibroblasts, MEFs) and mammalian animals (mice) (in vivo). In this case, however, LACV NSs did not provide an advantage to the virus [110]. 

## 10. NSs from Vertebrate-Infecting Bunyaviruses in Their Arthropod Vectors

Vertebrate-infecting bunyaviruses, with the exception of hantaviruses, are transmitted by arthropod vectors, including arachnids (ticks) and insects (mosquitoes, phlebotomines and culicoid flies) where, similarly to tospoviruses, they replicate and establish a persistent infection [157]. During infection of the arthropod vector, vertebrate-infecting bunyaviruses are targeted by antiviral RNA silencing, as indicated by the production of bunyavirus-specific small RNAs during bunyavirus infection (Table 2) [107,109,111]. The importance of RNA silencing in modulating bunyavirus replication and the establishment of a persistent infection in arthropods is supported by the fact that, at least for phlebovirus RVFV, persistency is only achieved in cells with active Dicer-2-based RNA silencing [109]. Similar investigations are needed to verify whether this is general to other *Bunyaviridae* genera.

Still, little is known regarding the role of NSs during propagative transmission of vertebrate-infecting bunyaviruses in the arthropod vector. Analysis of BUNV and a recombinant NSs-deletion BUNV indicates that NSs is required for efficient replication in cell lines (including mosquito *Aedes albopictus* cell line U4.4), and infection with NSs-deletion virus in mosquito *Aedes aegypti* revealed lower titers and delayed dissemination to salivary glands when compared to wild-type virus, suggesting that in the absence of NSs the virus has difficulties in overcoming cellular defenses in the midgut [158]. Considering the role of RNA silencing in the midgut barrier [101,159], it can be speculated that BUNV NSs counteracts the RNA silencing component of the midgut barrier. BUNV NSs was also observed to be non-essential in mosquito cell lines with impaired RNA silencing, further supporting that this proteins may have RSS activity [158]. NSs from orthobunyavirus LACV and phlebovirus RVFV have also been analyzed to some extent regarding their effect on RNA silencing in arthropods (respectively, mosquito and tick cell lines), however in both cases suppression of silencing was not observed [110,135]. 

Altogether, available data indicate NSs is relevant during bunyavirus infection of arthropod vectors, however clear proof for presence (or absence) of RSS activity with NSs from vertebrate-infecting bunyaviruses in arthropods is still lacking. 

## 11. The Enigma of NSs: Questions and Perspectives

While bunyaviruses are targeted by antiviral RNA silencing in plants, vertebrates and arthropods, currently only the NSs from plant-infecting tospoviruses has been clearly shown to contain RNA silencing suppressor activity. Considering the close ancestral relation of bunyaviruses, as well as many structural and functional similarities, the lack of clear proof on suppression of RNA silencing by the NSs from vertebrate-infecting bunyaviruses in both arthropods and vertebrates remains a matter for debate.

In light of this it is important to highlight that the two reported (contradictory) studies on the effect of orthobunyavirus LACV NSs in mammalian RNA silencing made use of different experimental set ups to induce silencing and to express NSs. The first study by Soldan et al. [156], in which synthetic siRNAs were transfected into mammalian cells (human cell line 293T) already expressing LACV NSs (from plasmid constructs transfected 24 h prior transfection of siRNAs), likely has used the best conditions to test LACV NSs for the ability to suppress RNA silencing due to a couple of reasons. Firstly, NSs was expressed a priori, being readily available at the time when siRNAs were transfected and allowing it to directly interfere with RNA silencing. This strategy has earlier been successfully used to demonstrate RSS activity with viral proteins [160,161]. Secondly, transfection of RNA duplex molecules smaller than 30 bp activates only the RNA silencing pathway, and not the interferon pathway [162,163]. As such, a transfection with siRNAs (21 nt) will effectively trigger RNA silencing only, and therefore results obtained with LACV NSs solely reflect interference on RNA silencing, not on IFN-induced defense responses. Altogether, this supports the observations made by Soldan et al. on the presence of RSS activity with LACV NSs. The downside of the study by Soldan et al. is that it involves a transient system and this does not reflect an authentic viral infection. In the second, contradictory study by Blakqori et al. [110], infections were performed with LACV recombinant viruses that either contained or lacked NSs. However, during this study it was only assumed that LACV NSs does not interfere with mammalian silencing because viruses had similar growth patterns and titers regardless of NSs, but the effect of NSs on the siRNA profile was not investigated. This second study used MEFs, instead of the human 293T cells used in the first study. Therefore, based on this result it would be too premature to discard the possibility of RSS activity in LACV NSs, nor with other bunyaviral NSs proteins. Future studies should also take into consideration the effect of NSs on small RNA profiles. Considering the recent success in verifying antiviral RNA silencing in mammals [20], future investigations on NSs from vertebrate-infecting bunyaviruses would also benefit from using a similar approach with undifferentiated cells, in which the production of virus-derived siRNAs is stronger and the IFN response is lacking or reduced [20,21]. 

Another reason why studies on this point for the vertebrate-infecting bunyaviruses so far have remained unresolved is that in mammalian cells the IFN-pathway is a major antiviral mechanism, while antiviral silencing is still being debated by some [164] and might be more secondary or limited to undifferentiated cells or certain cell types [20]. In addition, NSs from vertebrate-infecting bunyaviruses might not present a strong suppressor of RNA silencing [164]. Within the insect vector, evidence points to a role of RNA silencing in the midgut barrier where it influences vector competence [100,101] and modulates a persistent viral infection as supported by the observation that persistent viruses become pathogenic if an RSS active against insect RNA silencing is co-expressed [95,104]. Although speculative, in case bunyavirus NSs would present a strong suppressor of arthropod RNA silencing, it could thus disrupt the equilibrium of a persistent infection and turn it into a pathogenic one that might be fatal to the arthropod. A low level of RSS activity might thus be preferred and, although maybe more difficult to proof experimentally, be sufficient to suppress RNA silencing in the midgut and support dissemination in the vector. Thus, whether vertebrate-infecting bunyaviruses truly need to counteract antiviral RNA silencing remains an issue that still requires further investigation and should not be ruled out. Based on the hypothesis that negative-sense RNA viruses may have their ancestry in arthropods [165], it is possible to speculate on the origin of NSs, which could have evolved as an adaptation to allow the ancestry insect-specific bunyavirus to cross and adapt to the plant or vertebrate secondary host. However, it is not possible to dismiss that NSs evolved initially in the ancestral arthropod host playing a role in modulation of arthropod antiviral RNA silencing. Further investigation of the several recently identified insect-specific bunyaviruses [166], some without a NSs ORF [167,168,169] while others seem to harbor a NSs (unknown function) [170], might shed light on the evolutionary history of bunyaviruses as well as on the role played by NSs.

In conclusion, NSs from bunyaviruses remains an enigmatic protein, being a virulence factor in different cellular environments. However, as reviewed above, its interference on antiviral RNA silencing is only clearly described for tospovirus infections in plants, while still being debated in arthropods and vertebrates. As new data are gathered on the relevance of RNA silencing during infection in mammals as well as during persistent infections in arthropods, this may eventually also contribute to a deeper understanding of how bunyaviruses possibly affect this antiviral defense mechanism. The advancement in deep-sequencing technologies and the use of undifferentiated cell lines may provide further tools to functionally analyze NSs during bunyavirus infection. Considering the importance of NSs for plant- and vertebrate-infecting bunyaviruses, understanding its role during infection and its modus operandi will remain a continuing challenge for many years to come. 

## Figures and Tables

**Table 1:** List of identified RNA silencing suppressors (RSS) from plant-infecting viruses, and the respective strategy used to suppress the RNA silencing machinery.

Genome	Family, Genus	Virus ^1^	RSS	Strategy
ssDNA	*Geminiviridae*, *Begomovirus*	TYLCV	**V2**	• Suppresses local silencing, interacts with SGS3 [25,26,27]
ssDNA	*Geminiviridae*, *Begomovirus*	TYLCCNV	**βC1**	• Interacts with an endogenous suppressor of silencing (rgsCAM) to repress RDR6 expression and secondary siRNA production [28]
(−)ssRNA	*Unassigned*, *Tenuivirus*	RHBV	**NS3**	• Size-dependently binds siRNA and miRNA, suppresses silencing in plant and insect (drosophila) [8,29]
(−)ssRNA	*Unassigned*, *Tenuivirus*	RSV	**NS3**	• Size-independently binds dsRNA (minimum size of 9 bp) [30], suppression of local and systemic silencing [31]
(−)ssRNA	*Bunyaviridae*, *Tospovirus*	TSWV	**NSs**	• Binds dsRNA (size independently) [32] • Suppression of local and systemic silencing [8,9,10]
(−)ssRNA	*Bunyaviridae*, *Tospovirus*	GBNV	**NSs**	• Suppression of local silencing [33]
(−)ssRNA	*Bunyaviridae*, *Tospovirus*	GRSV	**NSs**	• Binds dsRNA (size independently) [32] • Suppression of local and systemic silencing [10]
(−)ssRNA	*Bunyaviridae*, *Tospovirus*	TYRV	**NSs**	• Binds dsRNA (size dependently) [32] • Suppression of local and systemic silencing [10]
(−)ssRNA	*Rhabdoviridae*, *Cytorhabdovirus*	LNYV	**Phosphoprotein (P)**	• Local and systemic silencing suppression (weak) in plants, but not in insects. Shown to not prevent siRNA accumulation [34] • Suppresses RISC-mediated cleavage and RNA silencing amplification, interacts with proteins AGO (1, 2, 4), RDR6 and SGS3 [35]
(−)ssRNA	*Rhabdoviridae*, *Nucleorhabdovirus*	RYSV	**P6**	• Interferes with production of secondary siRNAs likely through interaction with RDR6. Suppresses systemic silencing, but NOT local silencing. Initial analysis does not show binding to siRNA [36]
(+)ssRNA	*Bromoviridae*, *Cucumovirus*	CMV	**2b**	• Binds siRNA (and to a lesser extent long dsRNA) [37] • AGO1 and AGO4 interaction [38,39,40,41] • AGO1 repression (via miR168 upregulation) [42] • Interacts with RDR proteins [43]
(+)ssRNA	*Bromoviridae*, *Cucumovirus*	TAV	**2b**	• Binds siRNA (size selectively) [44]
(+)ssRNA	*Closteroviridae*, *Crinivirus*	SPCSV	**Rnase3**	• Endonuclease activity cleaves siRNA resulting in silencing-inactive products [45,46]
(+)ssRNA	*Closteroviridae*, *Closterovirus*	BYV	**p21**	• Binds siRNA (size selectively) [47,48] • Interferes with the miRNA pathway [49,50]
(+)ssRNA	*Closteroviridae*, *Closterovirus*	CTV	**p20, p23, CP**	• p20 and p23 suppress local silencing [51] • p20 and CP suppress cell-to-cell spread of silencing [51] • p23 enhances viral accumulation and distribution in the plant host [52]
(+)ssRNA	*Luteoviridae*, *Enamovirus*	PEMV-1	**P0**	• Destabilization of AGO1 (mediated by an F-box-like domain), suppression of local and systemic silencing [53]
(+)ssRNA	*Luteoviridae*, *Polerovirus*	BWYV	**P0**	• Suppresses local but not systemic silencing [54] • Targets AGO proteins for degradation [55,56,57] through the autophagy pathway [58]
(+)ssRNA	*Luteoviridae*, *Polerovirus*	Other polero viruses:	**P0**	• CYDV, PLRV, CABYV: destabilization of AGO1 (mediated by F-box-like domain), with suppression of local but not systemic silencing (except PLRV P0, shown to suppress both) [53,56,59,60]. CLRDV and SCYLV: suppression of local but not systemic silencing [59,61]
(+)ssRNA	*Potyviridae*, *Ipomovirus*	SPMMV	**P1**	• Interacts with AGO1 component of RISC loaded with siRNA or miRNA. WG/GW motifs are essential for interaction with AGO1 and required for the silencing suppressor activity [62]
(+)ssRNA	*Potyviridae*, *Potyvirus*	TEV	**HC-Pro**	• Binds siRNA (size selectively) [47,48] • Inhibits 3′ methylation of si/miRNA [63,64] • AGO1 repression via miR168 upregulation [42]
(+)ssRNA	*Potyviridae*, *Potyvirus*	TuMV	**HC-Pro**	• Interferes with biogenesis of primary siRNA (effect dependent on interaction with RAV2) [65] • Interferes with miRNA pathway [49,50,66]
(+)ssRNA	*Potyviridae*, *Potyvirus*	ZYMV	**HC-Pro**	• Binds siRNA, interacts with and inhibits HEN1 [67,68,69]
(+)ssRNA	*Tombusviridae*, *Aureusvirus*	PoLV	**P14**	• Binds ds(RNA) size independently [47,70]
(+)ssRNA	*Tombusviridae*, *Carmovirus*	TCV	**P38**	• Binds dsRNA (size independently) [47] • Interferes with biogenesis of primary siRNA (effect dependent on interaction with RAV2) [65] • Targets (interacts with) AGO1 via GW-motifs [71] • AGO1 repression via miR168 upregulation [42] • Shifts DCL usage (enhances DCL1 levels, leading to consequential DCL3 and DCL4 downregulation) [71]
(+)ssRNA	*Tombusviridae*, *Tombusvirus*	CIRV	**P19**	• Binds siRNA size selectively [48,72,73] • Inhibits the 3′ methylation of siRNAs [63] • AGO1 repression (via miR168 upregulation) [74]
(+)ssRNA	*Tombusviridae*, *Tombusvirus*	CymRSV	**P19**	• Binds siRNA size dependently [47,75] • AGO1 repression (via miR168 upregulation) [42,76]
(+)ssRNA	*Tombusviridae*, *Tombusvirus*	TBSV	**P19**	• Interferes with the miRNA pathway [49,50]
(+)ssRNA	*Virgaviridae*, *Hordeivirus*	BSMV	**γB**	• Binds siRNA size selectively [47]
(+)ssRNA	*Virgaviridae*, *Pecluvirus*	PCV	**P15**	• Binds siRNA size selectively [47]
(+)ssRNA	*Virgaviridae*, *Tobamovirus*	TMV	**p122**	• Binds siRNA and miRNA and interferes with HEN-1 mediated 3′ methylation of small RNAs [77] • AGO1 repression (via miR168 upregulation) [42]
(+)ssRNA	*Virgaviridae*, *Tobamovirus*	TMV	**p126**	• Interferes with HEN-1 mediated 3′ methylation of small RNAs [78] • Suppression of local and systemic silencing [79]
dsDNA(RT)	*Caulimoviridae*, *Caulimovirus*	CaMV	**Decoy RNA**	• Massive amounts of siRNAs are generated from a noncoding RNA (ncRNA) leader region, programming antiviral RNA silencing against this region and preventing effective targeting of the remaining genome [23]
dsDNA(RT)	*Caulimoviridae*, *Caulimovirus*	CaMV	**P6**	• Interaction with and inactivation of DRB4, and suppression of local silencing [80,81]

^1^ BSMV (barley stripe mosaic virus), BWYV (beet western yellows virus), BYV (beet yellows virus), CABYV (cucurbit aphid-borne yellows virus), CaMV (cauliflower mosaic virus), CIRV (carnation Italian ringspot virus), CLRDV (cotton leaf roll dwarf virus), CMV (cucumber mosaic virus), CTV (citrus tristeza virus), CYDV (cereal yellow dwarf virus), CymRSV (cymbidium ringspot virus), GBNV (groundnut bud necrosis virus), GRSV (groundnut ringspot virus), LNYV (lettuce necrotic yellows virus), PCV (peanut clump virus), PEMV-1 (pea enation mosaic virus-1), PLRV (potato leafroll virus), PoLV (pothos latent virus), RHBV (rice hoja blanca virus), RSV (rice stripe virus), RYSV (rice yellow stunt virus), SCYLV (sugarcane yellow leaf virus), SPCSV (sweet potato chlorotic stunt virus), SPMMV (sweet potato mild mottle virus), TAV (tomato aspermy virus), TBSV (tomato bushy stunt virus), TCV (turnip crinkle virus), TEV (tobacco etch virus), TMV (tobacco mosaic virus), TSWV (tomato spotted wilt virus), TuMV (turnip mosaic virus), TYLCCNV (tomato yellow leaf curl China virus), TYLCV (tomato yellow leaf curl virus), TYRV (tomato yellow ring virus), ZYMV (zucchini yellow mosaic virus).

**Table 2:** Reports on RNA silencing responses and production of virus-derived siRNAs during infection with members of *Bunyaviridae.*

Genus	Virus ^1^	Organism	Remarks	Ref.
*Orthobunyavirus*	LACV	**Insect (mosquito)** *Aedes albopictus* (C6/36 cell line) *Aedes triseriatus* (MAT cell line)	Northern-blot analysis of S RNA-derived mRNAs reveals presence of small RNAs mapping to these regions	[110]
		**Insect (mosquito and drosophila)** *Aedes albopictus* (C6/36 cell line) *Drosophila melanogaster* (S2 cell line)	Deep sequencing analysis shows presence of virus-specific small RNAs mapping to all three genomic segments	[111]
	SBV	**Insect (culicoides midges and mosquito)** *Aedes aegypti* (Aag2 cell line) *Culicoides sonorensis* (KC cell line)	Deep sequencing analysis shows presence of virus-specific small RNAs mapping to all three genomic segments	[107]
*Phlebovirus*	RVFV	**Insect (mosquito)** *Aedes aegypti* (Aag2 cell line) *Aedes albopictus* (C6/36 cell line) *Aedes albopictus* (U4.4 cell line)	Deep sequencing analysis shows presence of virus-specific small RNAs mapping to all three genomic segments. Results indicate antiviral RNA silencing is related to modulation of bunyavirus persistent infection in arthropods.	[109]
*Tospovirus*	TSWV	**Plant** *Nicotiana benthamiana*	Northern-blot analysis shows presence of virus-specific small RNAs mapping to all three genomic segments and a closer analysis of the S RNA show small RNAs mapping to most of its sequence, but hardly to the IGR region.	[112]
	TSWV PolRSV	**Plant** *Nicotiana benthamiana Solanum lycopersicum*	Deep sequencing analysis shows presence of virus-specific small RNAs mapping to all three genomic segments, but hardly to the IGR regions (from both S and M segments).	[113,114,115]

**^1^** LACV (La Crosse virus), PolRSV (polygonum ringspot virus), RVFV (Rift Valley fever virus), SBV (Schmallenberg virus), TSWV (tomato spotted wilt virus).

## References

[1] Jaaskelainen K.M., Kaukinen P., Minskaya E.S., Plyusnina A., Vapalahti O., Elliott R.M., Weber F., Vaheri A., Plyusnin A. (2007). Tula and Puumala hantavirus NSs ORFs are functional and the products inhibit activation of the interferon-beta promoter. J. Med. Virol..

[2] Kormelink R., Garcia M.L., Goodin M., Sasaya T., Haenni A.L. (2011). Negative-strand RNA viruses: The plant-infecting counterparts. Virus Res..

[3] King A.M.Q., Adams M.J., Carstens E.B., Lefkowitz E.J. (2012). Ninth Report of the International Committee on Taxonomy of Viruses.

[4] Bente D.A., Forrester N.L., Watts D.M., McAuley A.J., Whitehouse C.A., Bray M. (2013). Crimean-Congo hemorrhagic fever: History, epidemiology, pathogenesis, clinical syndrome and genetic diversity. Antiviral Res..

[5] Elliott R.M. (2014). Orthobunyaviruses: recent genetic and structural insights. Nat. Rev. Microbiol..

[6] Elliott R.M., Brennan B. (2014). Emerging phleboviruses.

[7] Eifan S., Schnettler E., Dietrich I., Kohl A., Blomstrom A.L. (2013). Non-structural proteins of arthropod-borne bunyaviruses: Roles and functions. Viruses.

[8] Bucher E., Sijen T., De Haan P., Goldbach R., Prins M. (2003). Negative-strand tospoviruses and tenuiviruses carry a gene for a suppressor of gene silencing at analogous genomic positions. J. Virol..

[9] Takeda A., Sugiyama K., Nagano H., Mori M., Kaido M., Mise K., Tsuda S., Okuno T. (2002). Identification of a novel RNA silencing suppressor, NSs protein of Tomato spotted wilt virus. FEBS Lett..

[10] Hedil M., Sterken M.G., de Ronde D., Lohuis D., Kormelink R. (2015). Analysis of Tospovirus NSs Proteins in Suppression of Systemic Silencing. PLoS ONE.

[11] Molnar A., Csorba T., Lakatos L., Varallyay E., Lacomme C., Burgyan J. (2005). Plant virus-derived small interfering RNAs originate predominantly from highly structured single-stranded viral RNAs. J. Virol..

[12] Bernstein E., Caudy A.A., Hammond S.M., Hannon G.J. (2001). Role for a bidentate ribonuclease in the initiation step of RNA interference. Nature.

[13] Hamilton A.J., Baulcombe D.C. (1999). A species of small antisense RNA in posttranscriptional gene silencing in plants. Science.

[14] Lee Y.S., Nakahara K., Pham J.W., Kim K., He Z., Sontheimer E.J., Carthew R.W. (2004). Distinct roles for Drosophila Dicer-1 and Dicer-2 in the siRNA/miRNA silencing pathways. Cell.

[15] Kim Y.J., Maizel A., Chen X. (2014). Traffic into silence: Endomembranes and post-transcriptional RNA silencing. EMBO J..

[16] Wassenegger M., Krczal G. (2006). Nomenclature and functions of RNA-directed RNA polymerases. Trends Plant Sci..

[17] Barnard A.C., Nijhof A.M., Fick W., Stutzer C., Maritz-Olivier C. (2012). RNAi in Arthropods: Insight into the Machinery and Applications for Understanding the Pathogen-Vector Interface. Genes.

[18] Pumplin N., Voinnet O. (2013). RNA silencing suppression by plant pathogens: Defence, counter-defence and counter-counter-defence. Nat. Rev. Microbiol..

[19] Bologna N.G., Voinnet O. (2014). The diversity, biogenesis, and activities of endogenous silencing small RNAs in Arabidopsis. Annu Rev. Plant Biol..

[20] Maillard P.V., Ciaudo C., Marchais A., Li Y., Jay F., Ding S.W., Voinnet O. (2013). Antiviral RNA interference in mammalian cells. Science.

[21] Li Y., Lu J., Han Y., Fan X., Ding S.W. (2013). RNA interference functions as an antiviral immunity mechanism in mammals. Science.

[22] Schwartz M., Chen J., Janda M., Sullivan M., den Boon J., Ahlquist P. (2002). A positive-strand RNA virus replication complex parallels form and function of retrovirus capsids. Mol. Cell.

[23] Blevins T., Rajeswaran R., Aregger M., Borah B.K., Schepetilnikov M., Baerlocher L., Farinelli L., Meins F., Hohn T., Pooggin M.M. (2011). Massive production of small RNAs from a non-coding region of Cauliflower mosaic virus in plant defense and viral counter-defense. Nucleic Acids Res..

[24] Csorba T., Kontra L., Burgyan J. (2015). viral silencing suppressors: Tools forged to fine-tune host-pathogen coexistence. Virology.

[25] Glick E., Zrachya A., Levy Y., Mett A., Gidoni D., Belausov E., Citovsky V., Gafni Y. (2008). Interaction with host SGS3 is required for suppression of RNA silencing by tomato yellow leaf curl virus V2 protein. Proc. Natl. Acad. Sci. USA.

[26] Fukunaga R., Doudna J.A. (2009). dsRNA with 5′ overhangs contributes to endogenous and antiviral RNA silencing pathways in plants. EMBO J..

[27] Zrachya A., Glick E., Levy Y., Arazi T., Citovsky V., Gafni Y. (2007). Suppressor of RNA silencing encoded by Tomato yellow leaf curl virus-Israel. Virology.

[28] Li F., Huang C., Li Z., Zhou X. (2014). Suppression of RNA silencing by a plant DNA virus satellite requires a host calmodulin-like protein to repress RDR6 expression. PLoS Pathog..

[29] Hemmes H., Lakatos L., Goldbach R., Burgyan J., Prins M. (2007). The NS3 protein of Rice hoja blanca tenuivirus suppresses RNA silencing in plant and insect hosts by efficiently binding both siRNAs and miRNAs. RNA.

[30] Shen M., Xu Y., Jia R., Zhou X., Ye K. (2010). Size-independent and noncooperative recognition of dsRNA by the Rice stripe virus RNA silencing suppressor NS3. J. Mol. Biol..

[31] Xiong R., Wu J., Zhou Y., Zhou X. (2009). Characterization and subcellular localization of an RNA silencing suppressor encoded by Rice stripe tenuivirus. Virology.

[32] Schnettler E., Hemmes H., Huismann R., Goldbach R., Prins M., Kormelink R. (2010). Diverging affinity of tospovirus RNA silencing suppressor proteins, NSs, for various RNA duplex molecules. J. Virol..

[33] Goswami S., Sahana N., Pandey V., Doblas P., Jain R.K., Palukaitis P., Canto T., Praveen S. (2012). Interference in plant defense and development by non-structural protein NSs of Groundnut bud necrosis virus. Virus Res..

[34] Mann K.S., Johnson K.N., Dietzgen R.G. (2015). Cytorhabdovirus phosphoprotein shows RNA silencing suppressor activity in plants, but not in insect cells. Virology.

[35] Mann K.S., Johnson K.N., Carroll B.J., Dietzgen R.G. (2016). Cytorhabdovirus P protein suppresses RISC-mediated cleavage and RNA silencing amplification in planta. Virology.

[36] Guo H., Song X., Xie C., Huo Y., Zhang F., Chen X., Geng Y., Fang R. (2013). Rice yellow stunt rhabdovirus protein 6 suppresses systemic RNA silencing by blocking RDR6-mediated secondary siRNA synthesis. Mol. Plant-Microbe Interact..

[37] Goto K., Kobori T., Kosaka Y., Natsuaki T., Masuta C. (2007). Characterization of silencing suppressor 2b of cucumber mosaic virus based on examination of its small RNA-binding abilities. Plant Cell Physiol..

[38] Zhang X., Yuan Y.R., Pei Y., Lin S.S., Tuschl T., Patel D.J., Chua N.H. (2006). Cucumber mosaic virus-encoded 2b suppressor inhibits Arabidopsis Argonaute1 cleavage activity to counter plant defense. Genes Dev..

[39] Gonzalez I., Martinez L., Rakitina D.V., Lewsey M.G., Atencio F.A., Llave C., Kalinina N.O., Carr J.P., Palukaitis P., Canto T. (2010). Cucumber mosaic virus 2b protein subcellular targets and interactions: Their significance to RNA silencing suppressor activity. Mol. Plant-Microbe Interact..

[40] Duan C.G., Fang Y.Y., Zhou B.J., Zhao J.H., Hou W.N., Zhu H., Ding S.W., Guo H.S. (2012). Suppression of Arabidopsis ARGONAUTE1-mediated slicing, transgene-induced RNA silencing, and DNA methylation by distinct domains of the Cucumber mosaic virus 2b protein. Plant Cell.

[41] Hamera S., Song X., Su L., Chen X., Fang R. (2012). Cucumber mosaic virus suppressor 2b binds to AGO4-related small RNAs and impairs AGO4 activities. Plant J..

[42] Varallyay E., Havelda Z. (2013). Unrelated viral suppressors of RNA silencing mediate the control of ARGONAUTE1 level. Mol. Plant Pathol..

[43] Diaz-Pendon J.A., Li F., Li W.X., Ding S.W. (2007). Suppression of antiviral silencing by cucumber mosaic virus 2b protein in Arabidopsis is associated with drastically reduced accumulation of three classes of viral small interfering RNAs. Plant Cell.

[44] Chen H.Y., Yang J., Lin C., Yuan Y.A. (2008). Structural basis for RNA-silencing suppression by Tomato aspermy virus protein 2b. EMBO Rep..

[45] Cuellar W.J., Kreuze J.F., Rajamaki M.L., Cruzado K.R., Untiveros M., Valkonen J.P. (2009). Elimination of antiviral defense by viral RNase III. Proc. Natl. Acad. Sci. USA.

[46] Weinheimer I., Jiu Y., Rajamaki M.L., Matilainen O., Kallijarvi J., Cuellar W.J., Lu R., Saarma M., Holmberg C.I., Jantti J. (2015). Suppression of RNAi by dsRNA-degrading RNaseIII enzymes of viruses in animals and plants. PLoS Pathog..

[47] Merai Z., Kerenyi Z., Kertesz S., Magna M., Lakatos L., Silhavy D. (2006). Double-stranded RNA binding may be a general plant RNA viral strategy to suppress RNA silencing. J. Virol..

[48] Lakatos L., Csorba T., Pantaleo V., Chapman E.J., Carrington J.C., Liu Y.P., Dolja V.V., Calvino L.F., Lopez-Moya J.J., Burgyan J. (2006). Small RNA binding is a common strategy to suppress RNA silencing by several viral suppressors. EMBO J..

[49] Chapman E.J., Prokhnevsky A.I., Gopinath K., Dolja V.V., Carrington J.C. (2004). Viral RNA silencing suppressors inhibit the microRNA pathway at an intermediate step. Genes Dev..

[50] Yu B., Chapman E.J., Yang Z., Carrington J.C., Chen X. (2006). Transgenically expressed viral RNA silencing suppressors interfere with microRNA methylation in Arabidopsis. FEBS Lett..

[51] Lu R., Folimonov A., Shintaku M., Li W.X., Falk B.W., Dawson W.O., Ding S.W. (2004). Three distinct suppressors of RNA silencing encoded by a 20-kb viral RNA genome. Proc. Natl. Acad. Sci. USA.

[52] Fagoaga C., Pensabene-Bellavia G., Moreno P., Navarro L., Flores R., Pena L. (2011). Ectopic expression of the p23 silencing suppressor of Citrus tristeza virus differentially modifies viral accumulation and tropism in two transgenic woody hosts. Mol. Plant Pathol..

[53] Fusaro A.F., Correa R.L., Nakasugi K., Jackson C., Kawchuk L., Vaslin M.F., Waterhouse P.M. (2012). The Enamovirus P0 protein is a silencing suppressor which inhibits local and systemic RNA silencing through AGO1 degradation. Virology.

[54] Pfeffer S., Dunoyer P., Heim F., Richards K.E., Jonard G., Ziegler-Graff V. (2002). P0 of beet Western yellows virus is a suppressor of posttranscriptional gene silencing. J. Virol..

[55] Csorba T., Lozsa R., Hutvagner G., Burgyan J. (2010). Polerovirus protein P0 prevents the assembly of small RNA-containing RISC complexes and leads to degradation of ARGONAUTE1. Plant J..

[56] Bortolamiol D., Pazhouhandeh M., Marrocco K., Genschik P., Ziegler-Graff V. (2007). The Polerovirus F box protein P0 targets ARGONAUTE1 to suppress RNA silencing. Curr. Biol..

[57] Baumberger N., Tsai C.H., Lie M., Havecker E., Baulcombe D.C. (2007). The Polerovirus silencing suppressor P0 targets ARGONAUTE proteins for degradation. Curr. Biol..

[58] Derrien B., Baumberger N., Schepetilnikov M., Viotti C., De Cillia J., Ziegler-Graff V., Isono E., Schumacher K., Genschik P. (2012). Degradation of the antiviral component ARGONAUTE1 by the autophagy pathway. Proc. Natl. Acad. Sci. USA.

[59] Delfosse V.C., Agrofoglio Y.C., Casse M.F., Kresic I.B., Hopp H.E., Ziegler-Graff V., Distefano A.J. (2014). The P0 protein encoded by cotton leafroll dwarf virus (CLRDV) inhibits local but not systemic RNA silencing. Virus Res..

[60] Pazhouhandeh M., Dieterle M., Marrocco K., Lechner E., Berry B., Brault V., Hemmer O., Kretsch T., Richards K.E., Genschik P. (2006). F-box-like domain in the polerovirus protein P0 is required for silencing suppressor function. Proc. Natl. Acad. Sci. USA.

[61] Mangwende T., Wang M.L., Borth W., Hu J., Moore P.H., Mirkov T.E., Albert H.H. (2009). The P0 gene of Sugarcane yellow leaf virus encodes an RNA silencing suppressor with unique activities. Virology.

[62] Giner A., Lakatos L., Garcia-Chapa M., Lopez-Moya J.J., Burgyan J. (2010). Viral protein inhibits RISC activity by argonaute binding through conserved WG/GW motifs. PLoS Pathog..

[63] Lozsa R., Csorba T., Lakatos L., Burgyan J. (2008). Inhibition of 3' modification of small RNAs in virus-infected plants require spatial and temporal co-expression of small RNAs and viral silencing-suppressor proteins. Nucleic Acids Res..

[64] Ebhardt H.A., Thi E.P., Wang M.B., Unrau P.J. (2005). Extensive 3′ modification of plant small RNAs is modulated by helper component-proteinase expression. Proc. Natl. Acad. Sci. USA.

[65] Endres M.W., Gregory B.D., Gao Z., Foreman A.W., Mlotshwa S., Ge X., Pruss G.J., Ecker J.R., Bowman L.H., Vance V. (2010). Two plant viral suppressors of silencing require the ethylene-inducible host transcription factor RAV2 to block RNA silencing. PLoS Pathog..

[66] Kasschau K.D., Xie Z., Allen E., Llave C., Chapman E.J., Krizan K.A., Carrington J.C. (2003). P1/HC-Pro, a viral suppressor of RNA silencing, interferes with Arabidopsis development and miRNA unction. Dev. Cell.

[67] Jamous R.M., Boonrod K., Fuellgrabe M.W., Ali-Shtayeh M.S., Krczal G., Wassenegger M. (2011). The helper component-proteinase of the Zucchini yellow mosaic virus inhibits the Hua Enhancer 1 methyltransferase activity in vitro. J. Gen. Virol..

[68] Shiboleth Y.M., Haronsky E., Leibman D., Arazi T., Wassenegger M., Whitham S.A., Gaba V., Gal-On A. (2007). The conserved FRNK box in HC-Pro, a plant viral suppressor of gene silencing, is required for small RNA binding and mediates symptom development. J. Virol..

[69] Fuellgrabe M.W., Boonrod K., Jamous R., Moser M., Shiboleth Y., Krczal G., Wassenegger M. (2011). Expression, purification and functional characterization of recombinant Zucchini yellow mosaic virus HC-Pro. Protein Expr. Purif..

[70] Merai Z., Kerenyi Z., Molnar A., Barta E., Valoczi A., Bisztray G., Havelda Z., Burgyan J., Silhavy D. (2005). Aureusvirus P14 is an efficient RNA silencing suppressor that binds double-stranded RNAs without size specificity. J. Virol..

[71] Azevedo J., Garcia D., Pontier D., Ohnesorge S., Yu A., Garcia S., Braun L., Bergdoll M., Hakimi M.A., Lagrange T. (2010). Argonaute quenching and global changes in Dicer homeostasis caused by a pathogen-encoded GW repeat protein. Genes Dev..

[72] Vargason J.M., Szittya G., Burgyan J., Hall T.M. (2003). Size selective recognition of siRNA by an RNA silencing suppressor. Cell.

[73] Rawlings R.A., Krishnan V., Walter N.G. (2011). Viral RNAi suppressor reversibly binds siRNA to outcompete Dicer and RISC via multiple turnover. J. Mol. Biol..

[74] Varallyay E., Olah E., Havelda Z. (2014). Independent parallel functions of p19 plant viral suppressor of RNA silencing required for effective suppressor activity. Nucleic Acids Res..

[75] Silhavy D., Molnar A., Lucioli A., Szittya G., Hornyik C., Tavazza M., Burgyan J. (2002). A viral protein suppresses RNA silencing and binds silencing-generated, 21- to 25-nucleotide double-stranded RNAs. EMBO J..

[76] Varallyay E., Valoczi A., Agyi A., Burgyan J., Havelda Z. (2010). Plant virus-mediated induction of miR168 is associated with repression of ARGONAUTE1 accumulation. EMBO J..

[77] Csorba T., Bovi A., Dalmay T., Burgyan J. (2007). The p122 subunit of Tobacco Mosaic Virus replicase is a potent silencing suppressor and compromises both small interfering RNA- and microRNA-mediated pathways. J. Virol..

[78] Vogler H., Akbergenov R., Shivaprasad P.V., Dang V., Fasler M., Kwon M.O., Zhanybekova S., Hohn T., Heinlein M. (2007). Modification of small RNAs associated with suppression of RNA silencing by tobamovirus replicase protein. J. Virol..

[79] Wang L.Y., Lin S.S., Hung T.H., Li T.K., Lin N.C., Shen T.L. (2012). Multiple Domains of the *Tobacco mosaic virus* p126 Protein Can Independently Suppress Local and Systemic RNA Silencing. Mol. Plant-Microbe Interact..

[80] Haas G., Azevedo J., Moissiard G., Geldreich A., Himber C., Bureau M., Fukuhara T., Keller M., Voinnet O. (2008). Nuclear import of CaMV P6 is required for infection and suppression of the RNA silencing factor DRB4. EMBO J..

[81] Laird J., McInally C., Carr C., Doddiah S., Yates G., Chrysanthou E., Khattab A., Love A.J., Geri C., Sadanandom A. (2013). Identification of the domains of cauliflower mosaic virus protein P6 responsible for suppression of RNA silencing and salicylic acid signalling. J. Gen. Virol..

[82] Li H., Li W.X., Ding S.W. (2002). Induction and suppression of RNA silencing by an animal virus. Science.

[83] Li H.W., Ding S.W. (2005). Antiviral silencing in animals. FEBS Lett.

[84] Garcia S., Billecocq A., Crance J.M., Munderloh U., Garin D., Bouloy M. (2005). Nairovirus RNA sequences expressed by a Semliki Forest virus replicon induce RNA interference in tick cells. J. Virol..

[85] Karlikow M., Goic B., Saleh M.C. (2014). RNAi and antiviral defense in Drosophila: Setting up a systemic immune response. Dev. Comp. Immunol..

[86] Okamura K., Ishizuka A., Siomi H., Siomi M.C. (2004). Distinct roles for Argonaute proteins in small RNA-directed RNA cleavage pathways. Genes Dev..

[87] Rand T.A., Ginalski K., Grishin N.V., Wang X. (2004). Biochemical identification of Argonaute 2 as the sole protein required for RNA-induced silencing complex activity. Proc. Natl. Acad. Sci. USA.

[88] Rand T.A., Petersen S., Du F.H., Wang X.D. (2005). Argonaute2 cleaves the anti-guide strand of siRNA during RISC activation. Cell.

[89] Bronkhorst A.W., van Rij R.P. (2014). The long and short of antiviral defense: Small RNA-based immunity in insects. Curr. Opin. Virol..

[90] Czech B., Hannon G.J. (2011). Small RNA sorting: Matchmaking for Argonautes. Nat. Rev. Genet..

[91] Li M.L., Weng K.F., Shih S.R., Brewer G. (2016). The evolving world of small RNAs from RNA viruses. Wiley Interdiscip. Rev. RNA.

[92] Chao J.A., Lee J.H., Chapados B.R., Debler E.W., Schneemann A., Williamson J.R. (2005). Dual modes of RNA-silencing suppression by Flock House virus protein B2. Nat. Struct Mol. Biol..

[93] Singh G., Popli S., Hari Y., Malhotra P., Mukherjee S., Bhatnagar R.K. (2009). Suppression of RNA silencing by Flock house virus B2 protein is mediated through its interaction with the PAZ domain of Dicer. FASEB J..

[94] Gammon D.B., Mello C.C. (2015). RNA interference-mediated antiviral defense in insects. Curr. Opin. Insect Sci..

[95] Nayak A., Berry B., Tassetto M., Kunitomi M., Acevedo A., Deng C., Krutchinsky A., Gross J., Antoniewski C., Andino R. (2010). Cricket paralysis virus antagonizes Argonaute 2 to modulate antiviral defense in Drosophila. Nat. Struct. Mol. Biol..

[96] Van Rij R.P., Saleh M.C., Berry B., Foo C., Houk A., Antoniewski C., Andino R. (2006). The RNA silencing endonuclease Argonaute 2 mediates specific antiviral immunity in Drosophila melanogaster. Genes Dev..

[97] Gray S.M., Banerjee N. (1999). Mechanisms of arthropod transmission of plant and animal viruses. Microbiol Mol. Biol. Rev..

[98] Hogenhout S.A., Ammar el D., Whitfield A.E., Redinbaugh M.G. (2008). Insect vector interactions with persistently transmitted viruses. Annu. Rev. Phytopathol..

[99] Franz A.W., Kantor A.M., Passarelli A.L., Clem R.J. (2015). Tissue Barriers to Arbovirus Infection in Mosquitoes. Viruses.

[100] Lan H., Chen H., Liu Y., Jiang C., Mao Q., Jia D., Chen Q., Wei T. (2015). Small Interfering RNA Pathway Modulates Initial Viral Infection in Midgut Epithelium of Insect after Ingestion of Virus. J. Virol..

[101] Khoo C.C., Piper J., Sanchez-Vargas I., Olson K.E., Franz A.W. (2010). The RNA interference pathway affects midgut infection- and escape barriers for Sindbis virus in Aedes aegypti. BMC Microbiol..

[102] Carissimo G., Pondeville E., McFarlane M., Dietrich I., Mitri C., Bischoff E., Antoniewski C., Bourgouin C., Failloux A.B., Kohl A. (2015). Antiviral immunity of Anopheles gambiae is highly compartmentalized, with distinct roles for RNA interference and gut microbiota. Proc. Natl. Acad. Sci. USA.

[103] Blair C.D. (2011). Mosquito RNAi is the major innate immune pathway controlling arbovirus infection and transmission. Future Microbiol..

[104] Cirimotich C.M., Scott J.C., Phillips A.T., Geiss B.J., Olson K.E. (2009). Suppression of RNA interference increases alphavirus replication and virus-associated mortality in Aedes aegypti mosquitoes. BMC Microbiol..

[105] Clarke B.D., Roby J.A., Slonchak A., Khromykh A.A. (2015). Functional non-coding RNAs derived from the flavivirus 3′ untranslated region. Virus Res..

[106] Siu R.W., Fragkoudis R., Simmonds P., Donald C.L., Chase-Topping M.E., Barry G., Attarzadeh-Yazdi G., Rodriguez-Andres J., Nash A.A., Merits A. (2011). Antiviral RNA interference responses induced by Semliki Forest virus infection of mosquito cells: Characterization, origin, and frequency-dependent functions of virus-derived small interfering RNAs. J. Virol..

[107] Schnettler E., Ratinier M., Watson M., Shaw A.E., McFarlane M., Varela M., Elliott R.M., Palmarini M., Kohl A. (2013). RNA interference targets arbovirus replication in Culicoides cells. J. Virol..

[108] Sabin L.R., Zheng Q., Thekkat P., Yang J., Hannon G.J., Gregory B.D., Tudor M., Cherry S. (2013). Dicer-2 Processes Diverse Viral RNA Species. PLoS ONE.

[109] Leger P., Lara E., Jagla B., Sismeiro O., Mansuroglu Z., Coppee J.Y., Bonnefoy E., Bouloy M. (2013). Dicer-2- and Piwi-mediated RNA interference in Rift Valley fever virus-infected mosquito cells. J. Virol..

[110] Blakqori G., Delhaye S., Habjan M., Blair C.D., Sanchez-Vargas I., Olson K.E., Attarzadeh-Yazdi G., Fragkoudis R., Kohl A., Kalinke U. (2007). La Crosse bunyavirus nonstructural protein NSs serves to suppress the type I interferon system of mammalian hosts. J. Virol..

[111] Brackney D.E., Scott J.C., Sagawa F., Woodward J.E., Miller N.A., Schilkey F.D., Mudge J., Wilusz J., Olson K.E., Blair C.D. (2010). C6/36 Aedes albopictus cells have a dysfunctional antiviral RNA interference response. PLoS Negl. Trop. Dis..

[112] Hedil M., Hassani-Mehraban A., Lohuis D., Kormelink R. (2014). Analysis of the A-U rich hairpin from the intergenic region of tospovirus S RNA as target and inducer of RNA silencing. PLoS ONE.

[113] Margaria P., Miozzi L., Rosa C., Axtell M.J., Pappu H.R., Turina M. (2015). Small RNA profiles of wild-type and silencing suppressor-deficient tomato spotted wilt virus infected Nicotiana benthamiana. Virus Res..

[114] Mitter N., Koundal V., Williams S., Pappu H. (2013). Differential expression of tomato spotted wilt virus-derived viral small RNAs in infected commercial and experimental host plants. PLoS ONE.

[115] Margaria P., Miozzi L., Ciuffo M., Rosa C., Axtell M.J., Pappu H.R., Turina M. (2015). Comparison of small RNA profiles in Nicotiana benthamiana and Solanum lycopersicum infected by polygonum ringspot tospovirus reveals host-specific responses to viral infection. Virus Res..

[116] Kormelink R., Kitajima E.W., De Haan P., Zuidema D., Peters D., Goldbach R. (1991). The nonstructural protein (NSs) encoded by the ambisense S RNA segment of tomato spotted wilt virus is associated with fibrous structures in infected plant cells. Virology.

[117] Hedil M., de Ronde D., Kormelink R. Biochemical analysis of NSs from different tospoviruses. Manuscript in preparation. NSs of TSWV, GRSV and TYRV biochemically analysed using electrophoretic mobility shift assays (EMSA) to investigate their affinity to small and long dsRNA.

[118] Vaucheret H., Vazquez F., Crete P., Bartel D.P. (2004). The action of ARGONAUTE1 in the miRNA pathway and its regulation by the miRNA pathway are crucial for plant development. Genes Dev..

[119] Moffett P. (2009). Mechanisms of recognition in dominant R gene mediated resistance. Adv. Virus Res..

[120] De Ronde D., Butterbach P., Kormelink R. (2014). Dominant resistance against plant viruses. Front. Plant Sci..

[121] Choi C.W., Qu F., Ren T., Ye X., Morris T.J. (2004). RNA silencing-suppressor function of Turnip crinkle virus coat protein cannot be attributed to its interaction with the Arabidopsis protein TIP. J. Gen. Virol..

[122] Wen R.H., Khatabi B., Ashfield T., Saghai Maroof M.A., Hajimorad M.R. (2013). The HC-Pro and P3 cistrons of an avirulent Soybean mosaic virus are recognized by different resistance genes at the complex Rsv1 locus. Mol. Plant-Microbe Interact..

[123] Angel C.A., Hsieh Y.C., Schoelz J.E. (2011). Comparative analysis of the capacity of tombusvirus P22 and P19 proteins to function as avirulence determinants in Nicotiana species. Mol. Plant-Microbe Interact..

[124] Tian Y.P., Valkonen J.P. (2013). Genetic determinants of Potato virus Y required to overcome or trigger hypersensitive resistance to PVY strain group O controlled by the gene Ny in potato. Mol. Plant-Microbe Interact..

[125] Zvereva A.S., Pooggin M.M. (2012). Silencing and Innate Immunity in Plant Defense Against Viral and Non-Viral Pathogens. Viruses.

[126] De Ronde D., Butterbach P., Lohuis D., Hedil M., van Lent J.W., Kormelink R. (2013). Tsw gene-based resistance is triggered by a functional RNA silencing suppressor protein of the Tomato spotted wilt virus. Mol. Plant Pathol..

[127] De Ronde D., Pasquier A., Ying S., Butterbach P., Lohuis D., Kormelink R. (2014). Analysis of Tomato spotted wilt virus NSs protein indicates the importance of the N-terminal domain for avirulence and RNA silencing suppression. Mol. Plant Pathol..

[128] Ding S.W. (2010). RNA-based antiviral immunity. Nat. Rev. Immunol..

[129] Badillo-Vargas I.E., Rotenberg D., Schneweis B.A., Whitfield A.E. (2015). RNA interference tools for the western flower thrips, Frankliniella occidentalis. J. Insect Physiol..

[130] Whitten M.M., Facey P.D., Del Sol R., Fernandez-Martinez L.T., Evans M.C., Mitchell J.J., Bodger O.G., Dyson P.J. (2016). Symbiont-mediated RNA interference in insects. Proc. Biol. Sci..

[131] Ullman D.E., German T.L., Sherwood J.L., Westcot D.M., Cantone F.A. (1993). Tospovirus replication in insect vector cells: Immunocytochemical evidence that the nonstructural protein encoded by the S RNA of tomato spotted wilt tospovirus is present in thrips vector cells. Phytopathology.

[132] Ullman D.E., Westcot D.M., Chenault K.D., Sherwood J.L., German T.L., Bandla M.D., Cantone F.A., Duer H.L. (1995). Compartmentalization, Intracellular Transport, and Autophagy of Tomato Spotted Wilt Tospovirus Proteins in Infected Thrips Cells. Phytopathology.

[133] Wijkamp I., van Lent J., Kormelink R., Goldbach R., Peters D. (1993). Multiplication of tomato spotted wilt virus in its insect vector, Frankliniella occidentalis. J. Gen. Virol..

[134] Margaria P., Bosco L., Vallino M., Ciuffo M., Mautino G.C., Tavella L., Turina M. (2014). The NSs protein of tomato spotted wilt virus is required for persistent infection and transmission by Frankliniella occidentalis. J. Virol..

[135] Garcia S., Billecocq A., Crance J.M., Prins M., Garin D., Bouloy M. (2006). Viral suppressors of RNA interference impair RNA silencing induced by a Semliki Forest virus replicon in tick cells. J. Gen. Virol..

[136] Oliveira V.C., Bartasson L., de Castro M.E., Correa J.R., Ribeiro B.M., Resende R.O. (2011). A silencing suppressor protein (NSs) of a tospovirus enhances baculovirus replication in permissive and semipermissive insect cell lines. Virus Res..

[137] De Oliveira V.C., da Silva Morgado F., Ardisson-Araujo D.M., Resende R.O., Ribeiro B.M. (2015). The silencing suppressor (NSs) protein of the plant virus Tomato spotted wilt virus enhances heterologous protein expression and baculovirus pathogenicity in cells and lepidopteran insects. Arch. Virol..

[138] Medeiros R.B., Resende Rde O., de Avila A.C. (2004). The plant virus Tomato Spotted Wilt Tospovirus activates the immune system of its main insect vector, Frankliniella occidentalis. J. Virol..

[139] Schneider W.M., Chevillotte M.D., Rice C.M. (2014). Interferon-stimulated genes: A complex web of host defenses. Annu. Rev. Immunol..

[140] Hoffmann H.H., Schneider W.M., Rice C.M. (2015). Interferons and viruses: An evolutionary arms race of molecular interactions. Trends Immunol..

[141] Billecocq A., Spiegel M., Vialat P., Kohl A., Weber F., Bouloy M., Haller O. (2004). NSs protein of Rift Valley fever virus blocks interferon production by inhibiting host gene transcription. J. Virol..

[142] Weber F., Bridgen A., Fazakerley J.K., Streitenfeld H., Kessler N., Randall R.E., Elliott R.M. (2002). Bunyamwera bunyavirus nonstructural protein NSs counteracts the induction of alpha/beta interferon. J. Virol..

[143] Hollidge B.S., Weiss S.R., Soldan S.S. (2011). The role of interferon antagonist, non-structural proteins in the pathogenesis and emergence of arboviruses. Viruses.

[144] Thomas D., Blakqori G., Wagner V., Banholzer M., Kessler N., Elliott R.M., Haller O., Weber F. (2004). Inhibition of RNA polymerase II phosphorylation by a viral interferon antagonist. J. Biol. Chem..

[145] Habjan M., Pichlmair A., Elliott R.M., Overby A.K., Glatter T., Gstaiger M., Superti-Furga G., Unger H., Weber F. (2009). NSs protein of rift valley fever virus induces the specific degradation of the double-stranded RNA-dependent protein kinase. J. Virol..

[146] Kalveram B., Lihoradova O., Ikegami T. (2011). NSs protein of rift valley fever virus promotes posttranslational downregulation of the TFIIH subunit p62. J. Virol..

[147] Rezelj V.V., Overby A.K., Elliott R.M. (2015). Generation of mutant Uukuniemi viruses lacking the nonstructural protein NSs by reverse genetics indicates that NSs is a weak interferon antagonist. J. Virol..

[148] Santiago F.W., Covaleda L.M., Sanchez-Aparicio M.T., Silvas J.A., Diaz-Vizarreta A.C., Patel J.R., Popov V., Yu X.J., Garcia-Sastre A., Aguilar P.V. (2014). Hijacking of RIG-I signaling proteins into virus-induced cytoplasmic structures correlates with the inhibition of type I interferon responses. J. Virol..

[149] Ning Y.J., Wang M., Deng M., Shen S., Liu W., Cao W.C., Deng F., Wang Y.Y., Hu Z., Wang H. (2014). Viral suppression of innate immunity via spatial isolation of TBK1/IKKepsilon from mitochondrial antiviral platform. J. Mol. Cell Biol..

[150] Chaudhary V., Zhang S., Yuen K.S., Li C., Lui P.Y., Fung S.Y., Wang P.H., Chan C.P., Li D., Kok K.H. (2015). Suppression of type I and type III interferon signalling by NSs protein of severe fever-with-thrombocytopenia syndrome virus through inhibition of STAT1 phosphorylation and activation. J. Gen. Virol..

[151] Ning Y.J., Feng K., Min Y.Q., Cao W.C., Wang M., Deng F., Hu Z., Wang H. (2015). Disruption of type I interferon signaling by the nonstructural protein of severe fever with thrombocytopenia syndrome virus via the hijacking of STAT2 and STAT1 into inclusion bodies. J. Virol..

[152] Wuerth J.D., Weber F. (2016). Phleboviruses and the Type I Interferon Response. Viruses.

[153] Walter C.T., Barr J.N. (2011). Recent advances in the molecular and cellular biology of bunyaviruses. J. Gen. Virol..

[154] Barnwal B., Karlberg H., Mirazimi A., Tan Y.J. (2016). The Non-structural Protein of Crimean-Congo Hemorrhagic Fever Virus Disrupts the Mitochondrial Membrane Potential and Induces Apoptosis. J. Biol. Chem..

[155] Bivalkar-Mehla S., Vakharia J., Mehla R., Abreha M., Kanwar J.R., Tikoo A., Chauhan A. (2011). Viral RNA silencing suppressors (RSS): Novel strategy of viruses to ablate the host RNA interference (RNAi) defense system. Virus Res..

[156] Soldan S.S., Plassmeyer M.L., Matukonis M.K., Gonzalez-Scarano F. (2005). La Crosse virus nonstructural protein NSs counteracts the effects of short interfering RNA. J. Virol..

[157] Horne K.M., Vanlandingham D.L. (2014). Bunyavirus-vector interactions. Viruses.

[158] Szemiel A.M., Failloux A.B., Elliott R.M. (2012). Role of Bunyamwera Orthobunyavirus NSs protein in infection of mosquito cells. PLoS Negl. Trop. Dis..

[159] Brackney D.E., Beane J.E., Ebel G.D. (2009). RNAi targeting of West Nile virus in mosquito midguts promotes virus diversification. PLoS Pathog..

[160] Schnettler E., Hemmes H., Goldbach R., Prins M. (2008). The NS3 protein of rice hoja blanca virus suppresses RNA silencing in mammalian cells. J. Gen. Virol..

[161] Li W.X., Li H., Lu R., Li F., Dus M., Atkinson P., Brydon E.W., Johnson K.L., Garcia-Sastre A., Ball L.A. (2004). Interferon antagonist proteins of influenza and vaccinia viruses are suppressors of RNA silencing. Proc. Natl. Acad. Sci. USA.

[162] Kim D.H., Behlke M.A., Rose S.D., Chang M.S., Choi S., Rossi J.J. (2005). Synthetic dsRNA Dicer substrates enhance RNAi potency and efficacy. Nat. Biotechnol..

[163] Siolas D., Lerner C., Burchard J., Ge W., Linsley P.S., Paddison P.J., Hannon G.J., Cleary M.A. (2005). Synthetic shRNAs as potent RNAi triggers. Nat. Biotechnol..

[164] Cullen B.R., Cherry S., tenOever B.R. (2013). Is RNA interference a physiologically relevant innate antiviral immune response in mammals?. Cell Host Microbe.

[165] Li C.X., Shi M., Tian J.H., Lin X.D., Kang Y.J., Chen L.J., Qin X.C., Xu J., Holmes E.C., Zhang Y.Z. (2015). Unprecedented genomic diversity of RNA viruses in arthropods reveals the ancestry of negative-sense RNA viruses. eLife.

[166] Bolling B.G., Weaver S.C., Tesh R.B., Vasilakis N. (2015). Insect-specific virus discovery: Significance for the arbovirus community. Viruses.

[167] Auguste A.J., Carrington C.V., Forrester N.L., Popov V.L., Guzman H., Widen S.G., Wood T.G., Weaver S.C., Tesh R.B. (2014). Characterization of a novel Negevirus and a novel Bunyavirus isolated from Culex (Culex) declarator mosquitoes in Trinidad. J. Gen. Virol..

[168] Marklewitz M., Handrick S., Grasse W., Kurth A., Lukashev A., Drosten C., Ellerbrok H., Leendertz F.H., Pauli G., Junglen S. (2011). Gouleako virus isolated from West African mosquitoes constitutes a proposed novel genus in the family Bunyaviridae. J. Virol..

[169] Marklewitz M., Zirkel F., Rwego I.B., Heidemann H., Trippner P., Kurth A., Kallies R., Briese T., Lipkin W.I., Drosten C. (2013). Discovery of a unique novel clade of mosquito-associated bunyaviruses. J. Virol..

[170] Marklewitz M., Zirkel F., Kurth A., Drosten C., Junglen S. (2015). Evolutionary and phenotypic analysis of live virus isolates suggests arthropod origin of a pathogenic RNA virus family. Proc. Natl. Acad. Sci. USA.

